# Past and future trends in cancer and biomedical research: a comparison between Egypt and the World using PubMed-indexed publications

**DOI:** 10.1186/1756-0500-5-349

**Published:** 2012-07-10

**Authors:** Ahmed Abdelmabood Zeeneldin, Fatma Mohamed Taha, Manar Moneer

**Affiliations:** 1 Medical Oncology/Hematology, National Cancer Institute, Cairo University, Fom El Khalig, Cairo, Egypt Zip Code 11796; 2 Medical Biochemistry, National Cancer Institute, Cairo University, Cairo, Egypt; 3 Biostatistics & Cancer Epidemiology, National Cancer Institute, Cairo University, Cairo, Egypt

**Keywords:** Egypt, PubMed, Biomedical research, Cancer research, Bibliometrics

## Abstract

**Background:**

PubMed is a free web literature search service that contains almost 21 millions of abstracts and publications with almost 5 million user queries daily. The purposes of the study were to compare trends in PubMed-indexed cancer and biomedical publications from Egypt to that of the world and to predict future publication volumes.

**Methods:**

The PubMed was searched for the biomedical publications between 1991 and 2010 (publications dates). Affiliation was then limited to Egypt. Further limitation was applied to cancer, human and animal publications. Poisson regression model was used for prediction of future number of publications between 2011 and 2020.

**Results:**

Cancer publications contributed 23% to biomedical publications both for Egypt and the world. Egyptian biomedical and cancer publications contributed about 0.13% to their world counterparts. This contribution was more than doubled over the study period. Egyptian and world’s publications increased from year to year with rapid rise starting the year 2003. Egyptian as well as world’s human cancer publications showed the highest increases. Egyptian publications had some peculiarities; they showed some drop at the years 1994 and 2002 and apart from the decline in the animal: human ratio with time, all Egyptian publications in the period 1991-2000 were significantly more than those in 2001-2010 (P < 0.05 for all). By 2020, Egyptian biomedical and cancer publications will increase by 158.7% and 280% relative to 2010 to constitute 0.34% and 0.17% of total PubMed publications, respectively.

**Conclusions:**

The Egyptian contribution to world’s biomedical and cancer publications needs significant improvements through research strategic planning, setting national research priorities, adequate funding and researchers’ training.

## Background

The size of the biomedical literature has grown exponentially over the past few years [1]. The PubMed is a database of publications and abstracts for biomedical literature in the fields of medicine, nursing, dentistry, veterinary medicine, health care systems, and preclinical sciences. It was developed and maintained by the National Center for Biotechnology Information (NCBI) [2]. In addition to the Medical Literature Analysis and Retrieval System Online (MEDLINE), PubMed provides access to old publications that are not converted to MEDLINE status, publications that precede the date of their journal were selected for MEDLINE indexing, publications for articles before Medical Subject Heading (MeSH) indexing and hence MEDLINE indexing, out-of-scope articles from certain MEDLINE journals and publications to some life science journals that are qualitatively reviewed by the National Library of medicine (NLM) [3]. The PubMed contains 24667 “only-PubMed” journal titles and 5591 journal titles “currently indexed for MEDLINE” as well as 8832 titles “previously indexed” and over time have ceased or changed titles [4,5]. As of 15 June 2011, there were over 20.9 million records indexed through the PubMed and the year 2010 witnessed the addition of more than 900000 new records (in the PubMed search box, type “1800:2100[dp]” or “2010[dp]”).

PubMed is a free Web literature search service and is the first choice first choice for electronically searching and retrieving biomedical literature. Almost 5 million queries are issued to PubMed each day by users around the globe [6], who rely on such access to keep abreast of the state of the art and make discoveries in their own fields [7]. Analysis of PubMed publications as an indicator of the research productivity of individual countries, regions or institutions has recently become a field of interest [8].

Cancer is a major worldwide health problem being one of the four leading threats to human health and development (along with cardiovascular diseases, chronic respiratory diseases and diabetes). In 2008, more than 12 million people were diagnosed and more than 7 million people died of cancer. In 2030, these figures will nearly double. Almost, 55% of new cancer cases and 65% of cancer deaths occur in the less developed world regions, including Egypt [9].

Egypt is one of the oldest civilizations in history and its contribution to human’s knowledge cannot be denied. Azhar is one of the oldest world’s universities that was settled in the 10th century and Cairo university being the current biggest Egyptian university was established in 1908 [10,11]. Four Egyptians won the Noble prize in Peace, Literature and Chemistry [12]. However, Egyptian current contribution to World’s biomedical publications seems low. Furthermore, there are no accessible comprehensive nation-wide publication databases through which all Egyptian literature can be traced accurately. The contribution of Egypt to the world’s biomedical publications in the PubMed increased from 0.09% in 1996 to 0.14% in 2006 [13] and over a decade (1992-2002), the quantitative growth of the Egyptian publications was 73% [14]. Egypt contributed about 17% of African articles and 30% of that of the Arab countries in the PubMed [15,16]. To the best of our knowledge, no reports had quantified the Egyptian cancer publications nor compared that to the Worlds’ figures of biomedical and cancer publications.

The aims of this study were to compare past trends in PubMed-indexed biomedical and cancer publications from Egypt to that of the entire world between 1991 and 2010 and to predict future trends in 2011 through 2020. The outcome of this study may alert researchers as well as decision makers in Egypt and similar countries to the current situation in biomedical and cancer research and the required level they should aim at.

## Methods

On the 25th of June 2011, the PubMed was searched using a methodology similar to that used in the literature [13,17,18]. The words “year: year[Date – Publication]” were typed in the research box. The word year was replaced by (1991:1991) through (2010:2010). This step retrieved the world’s total biomedical publications (WTBP) in the PubMed in the respective years. Then, the limits “Species = human” and “Species = animal” allowed retrieval of world’s human and animal biomedical publications (WHBP and WABP) in the PubMed, respectively. The limit “Subsets = Cancer” allowed retrieval of the world’s total cancer publications (WTBP) as well as the world’s human and animal cancer publications (WHCP and WACP) in the PubMed, respectively.

The above steps were then repeated with the affiliations limited to Egypt “Egypt[affiliation]”. This allowed retrieval of Egyptian total, human and animal biomedical publications in the PubMed (ETBP, EHBP and EABP, respectively) as well as Egyptian total, human and animal cancer publications (ETCP, EHCP and EACP, respectively).

Almost always, the total number of world’s publications was higher than the sum of its human and animal publications. This could be due to the presence of publications which could not be classified as animal or human (e.g. environmental research). Occasionally, the total number of Egyptian publications was lower than the sum of its human and animal publications. We manually revised the PubMed ID of these publications. Almost always, some publications had the same PubMed ID being classified as both animal and human at the same time. When this was encountered, they were reviewed and classified appropriately and the number of human or animal publications was changed accordingly. This happened for Egyptian human and animal cancer publications for the years 1991, 1992, 1995, 1996, 1999, and 2003 where 4, 7, 11, 4, 15 and 18 publications were classified as both human and animal. All were allocated to animal and removed from human publications of the respective years. Thus the number of human publications in the mentioned years is smaller than that the PubMed figures by a factor equal to the duplicate publications.

### Statistical analysis

Analyses were done using SPSS® software version 15 and Microsoft® excel 2007. Categorical variables were presented as percentage and group differences were assessed using Chi squared test. Numerical variables were presented as means/medians and standard deviations (SD)/interquartile ranges (IQR) Why not range. Means and medians were compared using the t-test or the Mann Whitney U test, respectively. A probability (two-sided) equal to or less than 0.05 was considered statistically significant.

The annual percent change (APC) was calculated by dividing the difference between the number of publications in a particular year and that of its preceding year by the latter and converting to a percentage. Considering the nature of the dependent variable (count of publications in the coming years), Poisson regression model was used for prediction of future number of publications between 2011 and 2020.

## Results

Between 1991 and 2010, there were 11 644 346 world biomedical publications listed in the PubMed (Table 1) with an annual mean (±SD) of 582 217 (±165 229). Of note, 2 696 136 publications were classified as being limited to the field of cancer (Table 2) with a mean (±SD) of 134 807 (±39 219). The percentage of cancer to total biomedical publications was almost stable (at ~23%) from 1991-2010. During the same period (1991-2010), there were 16835 biomedical publications listed in the PubMed with Egyptian affiliations with a median (IQR) of 668 (408-1096) (Table 1). Of note, 3928 publications (23.3%) were classified as being limited to the field of cancer with a median (IQR) of 125 (82-296) (Table 2). The percentage of Egyptian cancer to total biomedical publications increased from 16% in 1991 to 26% in 2010. Tables 1 and 2 shows the numbers of world’s and Egyptian publications retrieved from PubMed.

**Table 1:** Comparison between world’s and Egyptian biomedical publications listed in the PubMed database between 1991 and 2010

**Year**	**World’s biomedical publications**					**Egyptian biomedical publications**					**Egypt: world**		
	**TB**	**HB**	**HB:TB%**	**AB**	**AB:TB%**	**TB**	**HB**	**HB:TB%**	**AB**	**AB:TB%**	**TB%**	**HB%**	**AB%**
1991	407465	260956	64.0	110733	27.2	405	213	52.6	166	41.0	0.10	0.08	0.15
1992	412457	263753	63.9	111305	26.9	401	206	51.4	175	43.6	0.10	0.08	0.16
1993	420935	272842	64.8	113463	26.9	397	214	53.9	151	38.0	0.09	0.08	0.13
1994	431159	279339	64.8	117023	27.1	374	188	50.3	133	35.6	0.09	0.07	0.11
1995	441911	288261	65.2	118659	26.9	391	221	56.5	155	39.6	0.09	0.08	0.13
1996	451658	297744	65.9	119369	26.4	419	246	58.7	154	36.8	0.09	0.08	0.13
1997	450645	308457	68.5	121168	26.9	437	232	53.1	161	36.8	0.10	0.08	0.13
1998	468464	320319	68.4	124242	26.5	491	237	48.3	193	39.3	0.10	0.07	0.16
1999	486748	331268	68.1	125892	25.9	564	335	59.4	203	36.0	0.12	0.10	0.16
2000	527674	347170	65.8	132827	25.2	608	298	49.0	181	29.8	0.12	0.09	0.14
2001	542749	359194	66.2	135726	25.0	745	333	44.7	248	33.3	0.14	0.09	0.18
2002	559756	371142	66.3	140457	25.1	727	329	45.3	218	29.9	0.13	0.09	0.16
2003	590045	393435	66.7	146235	24.8	852	431	50.6	266	31.2	0.14	0.11	0.18
2004	634148	417840	65.9	154403	24.4	863	497	57.6	239	27.7	0.14	0.12	0.15
2005	694313	452729	65.2	165928	23.9	993	539	54.3	267	26.9	0.14	0.12	0.16
2006	739792	483368	65.3	178070	24.1	1131	607	53.7	296	26.1	0.15	0.13	0.17
2007	777249	507301	65.3	183230	23.6	1364	744	54.5	347	25.4	0.18	0.15	0.19
2008	825239	534925	64.8	190004	23.0	1564	865	55.3	374	23.9	0.19	0.16	0.20
2009	863722	552265	63.9	195160	22.6	1871	1042	55.7	465	24.8	0.22	0.19	0.24
2010	918217	526581	57.4	186026	20.3	2238	1128	50.4	483	21.8	0.24	0.21	0.26
Total	11644346	7568889	--	2869920	---	16835	8905	--	4875	--	--	--	--
Mean	582217	378444	65.3	143496	25.1	842	445	52.77	244	32.4	0.13	0.11	0.16
SD	165229	98951	2.3	29307	1.9	540	290	4.05	103	6.5	0.04	0.04	0.04

TB: number of total biomedical publications, HB: number of human biomedical publications, AB: number of animal biomedical publications, HB:TB%: human to total biomedical publications expressed as a percentage, AB:TB%: animal to total biomedical publications expressed as a percentage, TB%: Egyptian to world’s total biomedical publications expressed as percentage, HB%: Egyptian to world’s human biomedical publications expressed as percentage, AB%: Egyptian to world’s animal biomedical publications expressed as percentage, SD: standard deviation.

**Table 2:** Comparison between world’s and Egyptian cancer publications listed in the PubMed database between 1991 and 2010

**Year**	**World’s cancer publications**					**Egyptian cancer publications**					**Egypt: world**		
	**TC**	**HC**	**HC:TC%**	**AC**	**AC:TC%**	**TC**	**HC**	**HC:TC%**	**AC**	**AC:TC%**	**TC%**	**HC%**	**AC%**
1991	86770	65990	76.1	24003	27.7	67	43	64.2	21	31.3	0.08	0.07	0.09
1992	90268	68869	76.3	24895	27.6	81	50	61.7	26	32.1	0.09	0.07	0.10
1993	93822	72559	77.3	26358	28.1	76	48	63.2	23	30.3	0.08	0.07	0.09
1994	98468	76110	77.3	27777	28.2	75	50	66.7	23	30.7	0.08	0.07	0.08
1995	101286	78486	77.5	28937	28.6	81	45	55.6	29	35.8	0.08	0.06	0.10
1996	105241	82586	78.5	29743	28.3	86	57	66.3	26	30.2	0.08	0.07	0.09
1997	107363	84247	78.5	30227	28.2	83	64	77.1	19	22.9	0.08	0.08	0.06
1998	111551	88029	78.9	30841	27.7	106	61	57.5	43	40.6	0.10	0.07	0.14
1999	115159	91027	79.0	32197	27.9	120	70	58.3	43	35.8	0.10	0.08	0.13
2000	122177	96180	78.7	33815	27.7	117	72	61.5	44	37.6	0.10	0.07	0.13
2001	126058	100116	79.4	34713	27.5	136	84	61.8	48	35.3	0.11	0.08	0.14
2002	130928	104573	79.9	36238	27.7	129	82	63.6	42	32.6	0.10	0.08	0.12
2003	139492	112269	80.5	37568	26.9	188	123	65.4	56	29.8	0.13	0.11	0.15
2004	148903	119560	80.3	39903	26.8	215	167	77.6	46	21.4	0.14	0.14	0.12
2005	161317	132300	82.0	42635	26.4	252	198	78.6	49	19.4	0.16	0.03	0.11
2006	170712	138476	81.1	44875	26.3	311	247	79.4	63	20.3	0.18	0.18	0.14
2007	181512	147077	81.0	46885	25.8	321	240	74.7	67	20.9	0.18	0.16	0.14
2008	192402	155218	80.7	49064	25.5	409	289	70.6	84	20.5	0.21	0.19	0.17
2009	201089	160966	80.1	50845	25.3	485	341	70.3	116	23.9	0.24	0.21	0.23
2010	211618	156734	74.1	49894	23.6	590	384	65.1	130	22	0.28	0.25	0.26
Total	2696136	2131372	--	721413	--	3928	2715	--	998	--	--	--	--
Mean	134807	106569	78.9	36071	27.1	196	136	66.96	50	28.67	0.13	0.11	0.13
SD	39219	31721	1.9	8708	1.26	152	109	7.28	30	6.68	0.06	0.06	0.05

TC: number of total biomedical publications, HC: number of human biomedical publications, AC: number of animal biomedical publications, HC:TC%: human to total biomedical publications expressed as a percentage, AC:TC%: animal to total biomedical publications expressed as a percentage, TC%: Egyptian to world’s total cancer publications expressed as percentage, HC%: Egyptian to world’s human cancer publications expressed as percentage, AC%: Egyptian to world’s animal cancer publications expressed as percentage, SD: standard deviation.

Between 1991 and 2010, the mean contribution (±SD) of Egyptian biomedical publications to the worldwide PubMed publications was 0.13% (±0.04%). When classified into human and animal (Table 1), the figures were 0.11% (±0.04%) and 0.16% (±0.04%), respectively. The Egyptian contribution to biomedical publications increased by a factor of 2.4 from 1991 and 2010. Similarly, the Egyptian contribution to human and animal biomedical publications increased by factors of 2.6 and 1.7, respectively. The mean contribution (±SD) of Egyptian cancer publications to the worldwide cancer publications was 0.13% (±0.06%). When classified into human and animal, the figures were 0.11% (±0.06%) and 0.13% (±0.05%), respectively (Table 2). The Egyptian contribution to cancer publications increased by a factor of 3.5 from 1991 and 2010. Also, the Egyptian contribution to human and animal cancer publications increased by factors of 3.6 and 2.9, respectively.

**Figure 1:**
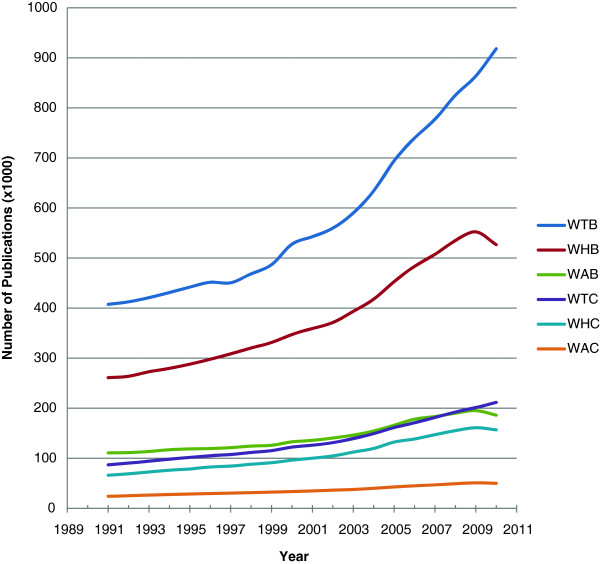
Numbers of World biomedical and cancer publications indexed in the PubMed between 1991 and 2010 (W: world, T: total, H: human, A: animal, B: biomedical, C: cancer).

**Figure 2:**
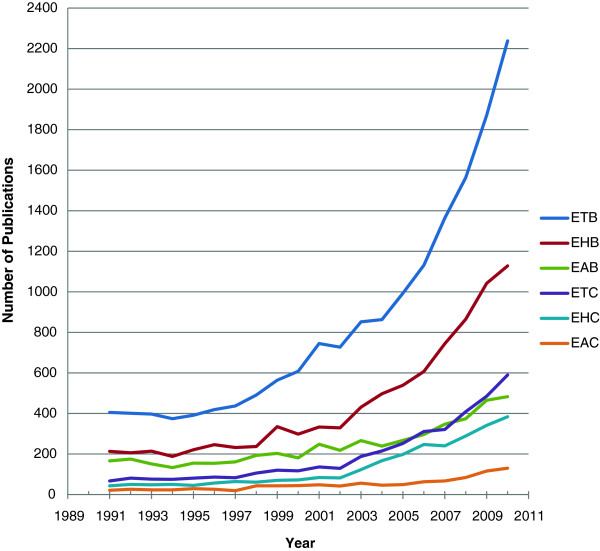
Numbers of Egyptian biomedical and cancer publications indexed in the PubMed between 1991 and 2010 (E: Egypt, T: total, H: human, A: animal, B: biomedical, C: cancer).

When plotting the World total biomedical publications indexed in PubMed between 1991 and 2010 (Figure 1), it is evident there is progressive rise in all types of publications. Furthermore, the pace of rise is more evident since the year 2003. Interestingly, cancer publications (all types) grew more than the biomedical publications. Also, there was a drop in the year 2010 in human and animal research whether it is biomedical or cancer related. Apart from slight drop in the years 1994 and 2002, Egyptian biomedical publications (total, human and animal) as well as cancer human publications showed continuous increase in numbers that is most marked in the most recent years particularly from the year 2005 onwards (Figure 2). However, cancer animal publications did not show the same pattern being almost stable throughout the evaluation period.

The study duration was divided into two periods; period 1 (1991-2000) and period 2 (2001-2010) (Table 3). For period 1, the Egyptian total biomedical publications were 4487 of which 892 (19.9%) were cancer related. The median of the Egyptian biomedical and cancer-related publications (IQR) were 412 (396-509) and 82 (76-109), respectively. For period 2, the Egyptian total biomedical publications were 12348 of which 2848 (23.1%) were cancer related. The median number of the biomedical and cancer-related publications (IQR) were 1062 (825-1640) and 282 (175-428), respectively. The increase in the animal biomedical publications (by a factor of 1.9) was less than the increase in the total and human biomedical publications increased (factors of 2.8, and 2.7, respectively) in period 2 compared to period 1. Similarly the increase in animal cancer publications (factor of 2.4) was less than that of total and human cancer publications (factors of 3.2 and 3.8, respectively) in period 2 compared to period 1. Apart from human biomedical publications expressed as a percentage of the total, all other categories were significantly higher in period 2 than period 1 (P < 0.05 for all).

**Table 3:** Comparisons of Egyptian biomedical and cancer Publications listed in the PubMed database over two time periods (1991-2000 & 2001-2010)

**Type of Publication**	**Period 1 (1991-2000)**			**Period 2 (2001-2010)**			**P value**
	**Total**	**Mean ± SD**	**median (IQR)**	**Total**	**Mean ± SD**	**Median (IQR)**	
Egyptian biomedical publications							
Total (n)	4487	449 ± 80	412 (396-509)	12348	1235 ± 515	1062 (825-1640)	<0.001*
Human (n)	2390	239 ± 44.8	226.5 (211-259)	6515	652 ± 284	573 (407-909)	<0.001*
Human: total (%)		53.3 ± 3.9	52.8 (50-57)		52.2 ± 4.4	54 (49-55)	0.56**
Animal (n)	1672	167.2 ± 21	163.5 (153-184)	3203	320 ± 94	282 (246-397)	<0.001**
Animal: total (%)		37.7 ± 3.7	37.4 (36-40)		27.1 ± 3.5	26.5 (25-30)	<0.001**
Egyptian cancer publications							
Total	892	89 ± 18	82 (76-109)	2848	304 ± 152	282 (175-428)	<0.001*
Human (n)	560	56 ± 10.4	53.5 (47-66)	2155	216 ± 104	219 (113-302)	<0.001*
Human: total ( %)		63.2 ± 6.1	62.4 (58-66)		70.7 ± 6.6	70.5 (65-78)	0.017**
Animal (n)	297	29.7 ± 9.8	26 (23-43)	701	70 ± 30.6	60 (48-92)	<0.001*
Animal: total (%)		32.7 ± 4.9	31.7 (30-36)		24.6 ± 5.8	21.7 (21-31)	0.003**
Egyptian cancer: biomedical publications (%)	19.9	19.8 ± 1.5	20.2 (19-21)	23.1	23.8 ± 3.4	25.1 (21-26)	<0.001**

SD: standard deviation, IQR: inter-quartile range, * Mann-Whitney U test, ** t-test.

Between 1991 and 2010, the annual percentage change (APC) of world biomedical publication ranged between -0.22% and 9.49% with a mean of 4.4% and a median of 3.95%. For the first (91-2000) and second (2001-2010) decades, the mean APCs were 2.94% and 6.03% while the median APCs were 2.43% and 6.17%, respectively. Also between 1991 and 2010, the APC of world cancer publications ranged between 2.02% and 8.34% with a mean of 4.82% and a median of 4.52%. For the first and second decades, the mean APCs were 3.88% and 5.93% while the median APCs were 3.90% and 6.00%, respectively. Between 1991 and 2010, the APC of Egyptian biomedical publication ranged between -5.79% and 22.53% with a mean of 9.75% and a median of 12.36%. For the first and second decades, the mean APCs were 4.81% and 13.28% while the median APCs were 4.55% and 15.06%, respectively. Also between 1991 and 2010, the APC of Egyptian cancer publications ranged between -45.59% and 45.74% with a mean of 12.94% and a median of 17.21%. For the first and second decades, the mean APCs were 12.48% and 12.93% while the median APCs were 13.21% and 21.65%, respectively.

**Figure 3:**
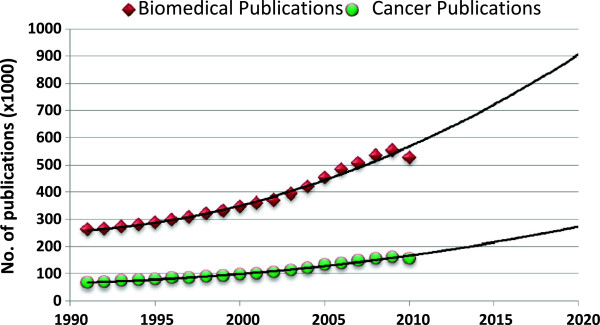
Past and future trends in world’s biomedical and cancer publications between 1991 and 2020.

For future prediction of number of publications between 2011 and 202, Poisson regression model was used. The regression equations for the total world and Egyptian publications were {log_e_ (Y) = -74.322 + 0.044*χ} and {log_e_ (Y) = -205.315 + 0.106*χ}, respectively. Assuming all conditions will remain the same, using these equations, the total world biomedical publications in 2020 are expected to mount to 857548 (95% CI: 855507-859595) publications with a 62.9% increase relative to 2010 (Figure 3). The Egyptian biomedical publications in 2020 are expected to mount to 2918 (95% CI: 2726-3124) publications with a 158.7% increase relative to 2010. In 2020, Egyptian biomedical publications will constitute 0.34% of the world’s figure.

**Figure 4:**
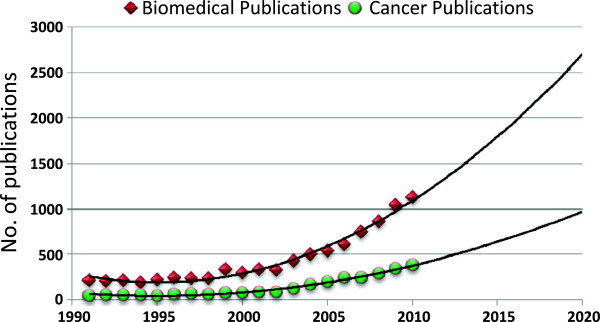
Past and future trends in Egyptian biomedical and cancer publications between 1991 and 2020.

The regression equations for the world and Egyptian cancer publications were {log_e_ (Y) = -88.510 + 0.050*χ} and {log_e_ (Y) = -296.166 + 0.137*χ}, respectively. Assuming all conditions will remain the same, using these equations, the world cancer publications in 2020 are expected to mount to 271171 (95%CI: 269962-272386) publications with a 73% increase relative to 2010. The Egyptian cancer publications in 2020 are expected to mount to 1459 (95%CI: 1288-1654) publications with a 280% increase relative to 2010 (Figure 4). In 2020, Egyptian Cancer publications will constitute 0.17% of the world’s figure.

## Discussion

Egypt is the “Land of Civilizations” and is reputed worldwide for its distinct 7,000-year-old record of civilization and immense wealth of knowledge [19]. Its 82-million population constitutes 1.2% of the total world’s population [20]. However, its contribution to the world’s biomedical research indexed in the PubMed is unexpectedly low being only 0.13%.This may not reflect accurately the total Egyptian publications as many of them may be published into PubMed non-indexed local journals that are available only in a printable form with no internet access. Language barriers may also hinder publishing Egyptian research [21]. One possible reason is the extremely low expenditure on research in Egypt that counts only to approximately 0.25% of the Gross Domestic Product (GDP) compared to 1.5-3% in developed countries [22]. The lack of researcher training is detrimental for research and subsequent publication. There is deficiency in the state-of-the art equipment. Moreover, not all the research institutions have clear research strategic plans [23]. Despite most of the Egyptian research budget is allocated to salaries of the administrators [21], Egyptian researchers are among the worst paid researchers in the Middle-East. The poor researchers mostly lack motivation [23]. Faced with lots of home-country difficulties, many excellent Egyptian researchers find their way in North America and Western Europe where they are welcomed and find good scientific atmosphere to perform high-end research that counts to these new countries. This brain drain deprives Egypt from the ability to build an advanced scientific community [21].

Our analysis showed that worldwide cancer publications account to almost one quarter of the total biomedical publications. This reflects the recognition of cancer as a major problem particularly in developed countries where cancer lies very high in top causes of mortality lists [24] as well as allocation of the needed resources as well as the strategic plans. The Egyptian cancer publications increased relative to total biomedical publications from 16% in 1991 to 26% in 2010. This could reflect the increasing awareness among Egyptian researchers of the cancer problem particularly the prevailing hepatocelluar and bladder carcinomas that are related to HCV endemic infection as well as the occupational exposure of farmers to Schistosoma mansoni [25]. This could also be explained by the ease in the availability of pathological applications and the study of biochemical markers that allow human-only research. Another reason could also be a shift in studying animals in relation to infectious disorders rather than to cancers as observed in the last decade.

Despite the progressive increase in publication numbers, world human biomedical and cancer publications represented fairly constant proportions of the total publications (~65% and 77%, respectively). The same was true for world animal biomedical and cancer publications (~25% for both). While the Egyptian human biomedical publications remained constant at ~50% of the total, that for cancer increased slightly from 64% to 70%. Egyptian animal biomedical publications showed progressive decline from 41% in 1991 to 22% in 2010. The same was also noted for animal cancer publications (31% in 1991 and 22% in 2010). The reasons of decline in Egyptian animal publications are unknown. However, limited availability particularly of the special strains, higher costs, the relative lack of animal breeding and housekeeping facilities coupled with a possible vanishing interest in animal research can possibly explain for such observation. Nevertheless, this should be investigated and corrective actions be taken as animal research can significantly save humans.

All types of world publications showed progressive increase with time. However this increase showed a higher pace starting the year 2003. The reason for this may be due to the progressive increase in research spending for life sciences particularly in the years 2001 and 2002 that is maintained thereafter [26]. Moreover, indexing coverage increased with time and the year 2003 witnessed the addition of 1.7 million old Medline citations to the PubMed database [27] World Biomedical and cancer human as well as animal publications showed decline in 2010. However, this observation may reflect that some publications may be still in the process of being added to the PubMed database. A late impact of the global financial crisis cannot be excluded completely. Follow up in a year or two may clarify this issue.

Egyptian publications showed progressive increase that was slight till the year 2002 when the rise was marked. While this can reflect a true rise, it can also reflect more publications in PubMed-indexed journals. The decline in Egyptian publications in the years 1994 and 2002 followed periods of economical difficulties [28,29] so that funding research that is almost completely state-dependent was determined.

Assuming all current conditions remain the same, it is expected that Egyptian biomedical and cancer publications will increase at a pace higher than that of the world. This will represent a catch-up phenomenon as the Egyptian publications are currently very low. To increase Egyptian publications from the current 0.24% to the desired 1.2% of the world’s publication to match the Egyptian/world’s total population, several actions have to be taken. Researchers move to PubMed-indexed and online journals can have rapid effects. Providing technical help for the so many local journals to be indexed as well as available on the internet will be also of help. Research strategic planning and setting national research priorities (e.g. HCV, HCC, Bilhariziasis) will be of great impact. Adequate funding of biomedical and cancer research with more participation of the private sector and the non-governmental organizations are eagerly needed. At the same time animal research needs to improve. Researchers’ training and awareness are of utmost importance. Setting animal breeding and housing facilities, importing and expanding unique animal strains will be of great help. Sharing equipment and adopting the concept of central research laboratories may also be of help.

The current study has some limitations. It was limited to PubMed indexed publications and the search was done on 25th June 2011. Any PubMed search repeated on a later day may yield different results, typically higher, as NLM may have processed more completed citations for various reasons, e.g., time lag in receipt, or a new journal for indexing going back to volume 1, or data from back issues such as those deposited in PubMed Central, or from other sources [30] The 19 million articles included in the PubMed do not represent the world total but rather a fraction that covers the period from 1950 to present. Only 30-80% of all known published randomized trials were identifiable using MEDLINE [31]. Relying exclusively on a MEDLINE search may retrieve a set of reports unrepresentative of all reports that would have been identified through a comprehensive search of several sources [32]. Databases, other than PubMed, are also available including Scopus, Web of Science and Google Scholar. Scopus covers a wider journal range includes more entries but with limited access to subscribers only. Web of Science have entries that date back to 1900 but with limited access to subscribers. Google Scholar presents all the benefits and drawbacks of the WWW [33].

Unequal indexing of publications by language and geography occurs across databases [34]. The PubMed considers many critical elements for a journal to be indexed. In addition, foreign language journals must contain an English-language abstract to be indexed. A journal may not be indexed being published for a local audience. Moreover, journal editors should submit an application to be indexed [35]. The current study did not identify many Egyptian articles that are not included in the PubMed databases. Thus, we believe that the Egyptian articles included in this review are lower than reality. However, there is no such an accessible and a comprehensive national database to quantify this sector. Another important aspect is that we identified Egyptian article through their affiliation to an Egyptian Institution. We could have missed entries that did not accurately affiliate and the work done by Egyptian researchers affiliated to non-Egyptian Institutions.

In conclusion, despite that Egyptian publications had increased markedly from 1991 to 2010, yet its contribution to the world’s overall publications needs be leveraged to match Egypt’s value as the “Land of Civilization”. Several actions need to be taken to achieve the desired research volumes.

## Competing interests

The authors declare no competing interests; financial or otherwise.

## Authors' contributions

All authors contributed to the work significantly. The first two authors developed the concept, searched the PubMed, collected data and wrote the manuscript. The first and third authors performed the statistical analyses and performed the literature search. All authors read and approved the final manuscript.

## References

[B1] Hunter L Cohen KB Biomedical language processing: what's beyond PubMed? Mol Cell 2006 21 589 594 10.1016/j.molcel.2006.02.012 16507357 PMC1702322

[B2] National Center for Biotechnology Information, US PubMed Help, Bethesda (MD) 2005 [online] available at: http://www.ncbi.nlm.nih.gov/books/NBK3830/ (accessed 15 June 2011)

[B3] National Library of Medicine, US Fact sheet; PubMed: MEDLINE Retrieval on the World Wide Web 2010 [online]. Available at: http://www.nlm.nih.gov/pubs/factsheets/pubmed.html (accessed 20 May, 2012)

[B4] National Library of Medicine, US List of Serials Indexed for Online Users 2012 [online]. Available at: http://www.nlm.nih.gov/tsd/serials/lsiou.html (accessed 20 May, 2012)

[B5] National Library of Medicine, US NLM catalog, journal subset 2012 [online]. Available at: http://www.ncbi.nlm.nih.gov/nlmcatalog/limits (accessed 20 May, 2012)

[B6] IslamajDogan R Murray GC Neveol A Understanding PubMed user search behavior through log analysis Database 2009 [online] available at: http://www.ncbi.nlm.nih.gov/pmc/articles/PMC2797455/?tool=pubmed (accessed 15 June) 10.1093/database/bap018 PMC2797455 20157491

[B7] Lu Z PubMed and beyond: a survey of web tools for searching literature Database 2011 (Oxford) 2011 Jan 18;2011:baq036. Print 2011. [online] available at: http://www.ncbi.nlm.nih.gov/pmc/articles/PMC3025693/ (accessed 15 June) 10.1093/database/baq036 PMC3025693 21245076

[B8] Tutarel O Geographical distribution of publications in the field of medical education BMC Med Educ 2002 2 1 3 10 10.1186/1472-6920-2-3 12031092 PMC113258

[B9] Ferlay J Shin HR Bray F Forman D Mathers C Parkin DM GLOBOCAN 2008 v1.2, Cancer Incidence and Mortality Worldwide: IARC CancerBase No. 10 2010 Lyon, France: International Agency for Research on Cancer [online]. Available from: http://globocan.iarc.fr. (Accessed 25 June 2012)

[B10] Azhar University History 2009 [online]. Available at: http://www.azhar.edu.eg/pages/history2.htm (accessed 20 May, 2012)

[B11] Cairo University About 2009 [online]. Available at: http://cuportal.cu.edu.eg/ (accessed 20 May, 2012)

[B12] Nobelprize.org All Nobel Prizes 2012 [online]. Available at: http://www.nobelprize.org/nobel_prizes/lists/all/ (accessed 15 June 2011)

[B13] Afifi M Egyptian Biomedical Publications in PubMed, 1996-2005 J Egypt Public Health Assoc 2007 82 1–2 91 104 18217326

[B14] Ghaleh NR Siadat F Azizi F Quantitative and qualitative assessment of biomedical publications from Iran, Pakistan and Egypt through their impact factor J Pak Med Assoc 2004 54 10 528 9 15552289

[B15] Uthman OA Uthman MB Geography of Africa biomedical publications: an analysis of 1996-2005 PubMed papers Int J Health Geogr 2007 6 46 56 10.1186/1476-072X-6-46 17927837 PMC2098756

[B16] Shaban SF Abu-Zidan FM A quantitative analysis of medical publications from Arab countries Saudi Med J 2003 24 3 294 6 12704508

[B17] Bissar-Tadmouri N Tadmouri GO Bibliometric analyses of biomedical research outputs in Lebanon and the United Arab Emirates (1988-2007) Saudi Med J 2009 30 1 130 9 19139787

[B18] Glynn RW Chin JZ Kerin MJ Sweeney KJ Representation of cancer in the medical literature--a bibliometric analysis. Representation of cancer in the medical literature--a bibliometric analysis PLoS One 2010 5 11 e13902 10.1371/journal.pone.0013902 21085482 PMC2976696

[B19] Egypt State Information Service Egypt: Land & People 2011 [online]. Available at: http://www.sis.gov.eg/En/Story.aspx?sid=1 (accessed 15 June 2011)

[B20] US Census Bureau U.S. & World Population Clocks 2010 [online]. Available at: http://www.census.gov/main/www/popclock.html (accessed 15 June 2011)

[B21] Aboulghar M Barriers to conducting clinical research in reproductive medicine: Egypt Fertil Steril 2011 96 4 805 6 10.1016/j.fertnstert.2011.08.044 21961913

[B22] World Bank Indicators 2011 [online]. Available at: http://data.worldbank.org/indicator/GB.XPD.RSDV.GD.ZS (accessed 15 June 2011)

[B23] Belal A Springuel I Research in Egyptian universities: the role of research in higher education Presentation at UNESCO Forum on Higher Education, Research and Knowledge, November 29 – December 1, 2006 [online]. Available at: http://portal.unesco.org/education/en/files/51625/11634283495Springuel-EN.pdf/Springuel-EN.pdf (accessed 15 June 2011)

[B24] Centers for Disease Control and Prevention Leading Causes of Death 2007 [online]. Available at: http://www.cdc.gov/nchs/fastats/lcod.htm (accessed 15 June 2011)

[B25] Gharbiah Population-based Cancer Registry Cancer Profile in Gharbiah-Egypt: Methodology and Results 1999 2002 The Middle East Cancer Consortium, Ministry of Health and Population, Egypt

[B26] National Science Foundation Science and engineering indicators 2010 [online]. Chapter available at: http://www.nsf.gov/statistics/seind10/c4/c4h.htm & figure available at: http://www.scientificamerican.com/article.cfm?id=graphic-science-funding (accessed 15 June 2011)

[B27] National Library of Medicine, US PubMed Celebrates its 10th Anniversary 2006 [online]. Available at: http://www.nlm.nih.gov/pubs/techbull/so06/so06_pm_10.html (accessed 20 May, 2012)

[B28] Rivilin P Egypt's demographic challenges and economic responses MERIA Journal 2003 7 4 [online] available at: http://www.gloria-center.org/meria/2003/12/rivlin.pdf (accessed 8 Feb 2013)

[B29] Ministry of Finance, Egypt Egyptian Economic Monitor, December 2010, volume II, No. 2 [Online] available at: http://www.mof.gov.eg/MOFGallerySource/English/PDF/Monitor_Dec_web.pdf (accessed 15 June 2011)

[B30] National Library of Medicine, US MEDLINE Citation Counts by Year of Publication 2012 [online]. Available at: http://www.nlm.nih.gov/bsd/medline_cit_counts_yr_pub.html (accessed 20 May, 2012)

[B31] Dickersin K Scherer R Lefebvre C Identifying relevant studies for systematic reviews BMJ 1994 309 6964 1286 91 10.1136/bmj.309.6964.1286 7718048 PMC2541778

[B32] Higgins JPT Green S Cochrane Handbook for Systematic Reviews of Interventions 2008 The Cochrane Collaboration [online]. Available at: http://www.cochrane-handbook.org (accessed 20 May 2012)

[B33] Falagas ME Pitsouni EI Malietzis GA Pappas G Comparison of PubMed, Scopus, Web of Science, and Google Scholar: strengths and weaknesses FASEB J 2008 22 2 338 42 17884971 10.1096/fj.07-9492LSF

[B34] Elsevier All Bibliographic Databases 2013 [online]. Available at: http://www.elsevier.com/bibliographic-databases (accessed 8 February 2013)

[B35] National Library of Medicine, US Fact Sheet; MEDLINE Journal Selection 2012 [online]. Available at: http://www.nlm.nih.gov/pubs/factsheets/jsel.html (accessed 20 May, 2012)

